# The Zika Pandemic - A Perfect Storm?

**DOI:** 10.1371/journal.pntd.0004589

**Published:** 2016-03-18

**Authors:** Philip K. Russell

**Affiliations:** Sabin Vaccine Institute, Washington, D.C., United States of America; Yale School of Public Health, UNITED STATES

The Zika virus, previously known as an uncommon zoonotic virus in Africa, has spread first to Southeast Asia and then to the Pacific islands and to the Americas causing a global health emergency due to the associated increase in children born with microcephaly and an increase in the incidence of Guillain-Barre syndrome. The explanation for what caused this virus, previously confined to a zoonotic niche in Africa, to become a major pandemic threat is as yet unknown but three factors are thought to play a significant role. It is probable that no single factor is responsible but the interaction of several factors has enabled this virus to spread across the globe. The increase in *Aedes aegypti* infestations throughout most of tropical and subtropical Americas certainly is an important factor and the spread of *Aedes albopictus* may play a role. However, *Aedes* vectors have been present in tropical and sub-tropical Americas in sufficient numbers to support dengue and chikungunya epidemics in the Americas for over forty years. An increase in vector prevalence alone does not explain the pandemic. Two other factors may play significant role.

The adaptation of the Zika virus to the human host by changes in codon usage in non-structural proteins has resulted in increased fitness of the virus for replication in the human host [1]. The pandemic strain of Zika differs significantly from the African strain in codon usage in the NS1 and NS4 regions of the genome. These acquired genetic changes increase its fitness for replication in the human host without changes in the protein sequence. Codon usage by the pandemic strain is optimized for replication in human cells. Codon optimization could result in higher viremias and increased infectivity for mosquito vectors. These synonymous mutations are an important evolutionary change in the virus related to the spread to the Pacific islands and the Americas

Another major factor is immune enhancement by pre-existing heterologous anti-flavivirus antibodies. Immune enhancement by non-neutralizing cross-reactive antibodies results in virus penetration of cells through the Fc receptor and increased viral replication. Immune enhancement plays a major role in the pathogenesis of severe dengue infections [2,3]. Zika virus replication in cell culture has been shown to be enhanced by heterologous flavivirus antibodies [4]. The current Zika pandemic is occurring in regions where dengue had been epidemic or is currently endemic so it is likely that pre-existing dengue immunity can cause increased virus replication in patients resulting in increased viremia and increased infectivity for vector mosquitos. Whether immune enhancement plays a role in the complications associated with Zika is a subject for future research.

The above factors proposed as major elements involved in promoting and sustaining the Zika pandemic are not mutually exclusive, indeed they can interact in a synergistic fashion. Both codon optimization and immune enhancement can make this virus a more effective human pathogen and more infectious to the vectors. Acting together they can create the virologic and epidemiologic equivalent of a perfect storm.
